# Is type III prostatitis also associated with bacterial infection?

**DOI:** 10.3389/fcimb.2023.1189081

**Published:** 2023-07-03

**Authors:** Wei-Jie Song, Jun Gao, Ji-Wei Huang, Yuan Liu, Zhi Long, Le-Ye He

**Affiliations:** ^1^ Department of Urology, The Third Xiangya Hospital, Central South University, Changsha, Hunan, China; ^2^ Sexual Health Research Center, The Third Xiangya Hospital, Central South University, Changsha, Hunan, China

**Keywords:** chronic prostatitis, expressed prostatic secretion, microbiology, 16s rRNA sequencing technique, metagenomics

## Abstract

**Objective:**

To explore whether type III prostatitis is related to bacterial infection by detecting the composition and function of microorganisms in expressed prostatic secretion (EPS) of patients with chronic prostatitis (CP) and healthy people.

**Methods:**

According to the inclusion and exclusion criteria, 57 subjects were included in our study, divided into the healthy group, type II prostatitis group, and type III prostatitis group. 16s rRNA sequencing technique was used to detect and analyze the microbial composition of EPS in each group. Additionally, the metagenomics sequencing technique was used to further explore the function of different bacteria in the type III prostatitis group. Data analysis was performed by bioinformatics software, and the results were statistically significant when P<0.05.

**Results:**

Many microorganisms exist in EPS in both CP patients and healthy populations. However, the relative abundance of *Pseudomonas*, *Haemophilus*, *Sneathia*, *Allobaculum*, and *Enterococcus* in CP patients (including type II and III) were significantly different. Still, the relative abundance of different bacteria in type II prostatitis patients was much higher than in type III. The metagenomics sequencing results for the type III prostatitis group showed that the different bacteria had certain biological functions.

**Conclusion:**

Based on our sequencing results and previous studies, we suggest that type III prostatitis may also be caused by bacterial infection.

## 1 Introduction

Prostatitis is a disease induced by various causes, with urethral irritation symptoms and chronic pelvic pain as the primary clinical manifestations, mostly in young and middle-aged men (Wang et al., 2018). Notably, prostatitis has a high incidence in China. Although it does not directly threaten the lives of patients, its clinical characteristics, which is difficult to treat and easy to relapse, can also seriously impact patients’ quality of life (Song et al., 2019; Yu and Gao, 2020). The National Institutes of Health (NIH) divides prostatitis into four types: acute type, chronic bacterial type, chronic non-bacterial type, and asymptomatic type, according to the length of the patient’s course and whether the bacteria can be detected in expressed prostatic secretion (EPS). Among them, chronic bacterial type (type II) and chronic non-bacterial type (type III) are collectively referred to as chronic prostatitis (CP), which is the most common clinical type of prostatitis in China (Krieger et al., 1999). Pathogenic bacteria can be detected in patients with type II prostatitis EPS. Thus, the cause of this is clear and can be treated symptomatically. Type III prostatitis is common in the clinic. However, its pathogenesis is unclear, and the therapeutic effect is not ideal because no pathogenic bacteria can be detected in the EPS of the patients by bacterial culture *in vitro* (Videcnik et al., 2015).

In recent years, with the continuous development and progress of microbial detection technology, researchers have found that many microorganisms exist in the human body, including pathogenic microorganisms and some opportunistic pathogens. These microorganisms not only participate in the process of material metabolism and immune regulation but also play an essential role in the maintenance of human health and the occurrence and development of diseases (Kong et al., 2022). Importantly, preliminary results show that although pathogenic bacteria can’t be detected in the EPS of type III prostatitis patients *in vitro*, infections of pathogenic microorganisms, such as mycoplasma, chlamydia, trichomonas, Candida, viruses, and parasites, may cause clinical symptoms of CP in patients (Skerk et al., 2002; Irajian et al., 2016; Arora et al., 2017).

Inspired by this, we aimed to detect and analyze the composition and functional characteristics of the microbial community in EPS of patients with CP (type II and III) compared with those of healthy individuals, using 16s rRNA gene sequencing and metagenomics techniques. Furthermore, we aimed to explore whether there is a correlation between type III prostatitis and bacterial infection.

## 2 Materials and methods

### 2.1 Study participants

According to the inclusion and exclusion criteria, we included 44 patients with CP and 13 healthy participants who underwent fertility examinations at Third Xiangya Hospital of Central South University from June 2022 to December 2022. All subjects were between the ages of 17 and 51. All patients included in the study underwent “two-glass test” (microscopic examination and bacterial culture of urine collected before and after prostate massage) in order to make a definite diagnosis and aid in the classification of prostatitis. According to the classification criteria of NIH, CP patients included in our study were divided into type II and type III prostatitis groups. Healthy people were designated as the control group (EPS and urine examination are both normal). The severity of the disease and mental state of the subjects were assessed by using The NIH chronic prostatitis symptom index (NIH-CPSI), Generalized Anxiexy Disorde-7 (GAD-7), and Patient Health Questionnaire-9 (PHQ-9) scales (**Supplementary Tables 1–3**). Our study was approved by the Ethics Committee of Third Xiangya Hospital of Central South University (Grant No. 22069), and the subjects’ informed consent was obtained.

All CP patients included in the study met the following inclusion criteria: (i) Were diagnosed with CP according to NIH classification and have the clinical symptoms for more than 3 months (Franco et al., 2019); (ii) did not take any CP therapy, including antibiotics, for at least 3 months before our study began; and (iii) Signed informed consent. Exclusion criteria include (i) urinary tract infection; (ii) history of urinary tumor, surgery, radiotherapy, systemic chemotherapy; (iii) unilateral testicular pain, active urethral stricture or bladder stone with pelvic symptoms, or any other urinary disease associated with lower urinary tract symptoms, any neurological disease or disorder affecting the bladder. (iv) took antimicrobial agents or other CP therapeutic drugs within 3 months before being included in our study; (v) did not sign informed consent.

Before collecting EPS samples, the subjects were asked to empty the urine in the bladder, clean the urethral orifice with sterile saline, and wipe the residual liquid near the urethral orifice with sterile gauze. The same urologist aseptically collected our study’s patient EPS samples. All specimens were stored in a refrigerator at -80°C immediately after collection.

### 2.2 16S rRNA gene sequencing analysis

Total genomic DNA was extracted from EPS samples using the Cetyltrimethylammonium Bromide (CTAB) method. The V3+V4 variable regions of the 16S ribosomal RNA (rRNA) gene was amplified by polymerase chain reaction (PCR) using 341F (5’-CCTAYGGGRBGCASCAG-3’) and 806R (5’-GGACTACNNGGGTATCTAAT-3’) primers. The PCR products were purified using 2% agarose gel electrophoresis in 1x TAE buffer, and bands of interest were excised. After equimolar pooling of purified PCR products, the Illumina TruSeq^®^ DNA PCR-Free Sample Preparation Kit (Illumina, USA) was used to construct the library, followed by index coding. The constructed library was quantified using Qubit and evaluated by library quality analysis. Finally, NovaSeq 6000 PE250 was used for sequencing to obtain paired-end reads of 250 bp. Then, the raw data was analyzed (DeSantis et al., 2006).

### 2.3 Metagenomic analysis

Total genomic DNA was extracted from EPS samples using the CTAB method. The purity and integrity of DNA were analyzed via agarose gel electrophoresis. Nanodrop was used to determine the purity of DNA (OD 260/280 ratio), and Qubit 2.0 was used to quantify DNA concentration accurately. After passing quality control, DNA samples were randomly fragmented using a Covaris ultrasonic disruptor. This was followed by end repair, A-tailing, adapter ligation, purification, and PCR amplification to complete the library preparation process. Following the completion of library construction, Qubit 2.0 was used for preliminary quantification, and the library was diluted and then analyzed for insertion fragment size using the Agilent 2100. Effective library concentration was accurately quantified using Q-PCR. After passing quality control, different libraries were pooled into a flowcell according to the required effective concentration and the target amount of sequencing data. Furthermore, the cBot was used for clustering, followed by Illumina PE150 (2x150) high-throughput sequencing platform for sequencing.

### 2.4 Bioinformatics analysis

The 16S rRNA gene sequencing analysis process was mainly completed following the “Atacama soil microbiome tutorial” in the Qiime2 documentation (https://docs.qiime2.org/2019.1/). The Qiime2 feature-classifier plugin was used to align the representative sequences of ASVs to the pre-trained 13_8 version 99% similarity GREENGENES database (trimmed to the V3V4 region based on the 341F/806R primers) to obtain a table of species classification information (Bokulich et al., 2018). All contaminating mitochondrial and chloroplast sequences were removed using the QIIME2 feature-table plugin. The multivariate statistical analysis method Partial Least Squares Discriminant Analysis (PLS-DA) was used with the R software package “mixOmics” to classify the research objects based on observed or measured variable values and reveal the relationship between the microbial community and sample categories (Love et al., 2014). The QIIME2 core-diversity plugin was used to calculate diversity matrices. Alpha diversity indices of feature sequence level, including observed OTUs, Chao index, Faith’s Phylogenetic Diversity, Shannon index, and Simpson index, were used to assess the level of sample diversity. Beta diversity indices, including Bray Curtis, unweighted UniFrac, and weighted UniFrac indices, were used to evaluate differences in microbial community structure between samples and were displayed using PCoA and NMDS plots (Vázquez-Baeza et al., 2019). After obtaining the overall beta diversity index, PERMANOVA, and ANOSIM methods were used to compare significant differences in the composition and structure of the microbial community among the different sample groups. Microbes with significant differences in abundance between groups and samples were identified using the LEfSe and DEseq2 methods (Mandal et al., 2015; Rohart et al., 2017). Metagenomics analysis displayed the Kyoto Encyclopedia of Genes and Genomes (KEGG) pathway categories, and KEGG Orthologous groups (KOs) detected using heatmap analysis. Linear discriminant analysis Effect Size (LEfSe) analysis was used to detect KEGG pathway categories and KOs in the EPS of CP patients and healthy controls. Those with LDA values > 2.0 and P < 0.05 were significantly enriched. Furthermore, pathway maps were used for pathway analysis, labeling each group’s detected genes and gene biomarkers. Unless otherwise specified, the parameters used in the above analysis were default settings.

## 3 Results

### 3.1 Characteristics of the study population

According to the inclusion, exclusion, and NIH classification criteria, 15 CP patients were included in the type II prostatitis group, 29 CP patients were included in the type III prostatitis group, and 13 healthy subjects were included in the control group. There was no significant difference in the basic characteristics of all subjects (**Table 1**), and the matching between groups was similar. Then, under the guidance of a male urologist, the NIH-CPSI, GAD-7, and PHQ-9 scales completed by the subjects were collected. Through the analysis of the results of the scales, it was found that the pain symptoms, lower urinary tract symptoms, and quality of life of CP patients were significantly worse than those of healthy people. At the same time, compared with healthy people, patients with any type of CP were accompanied by a certain degree of mental and psychological disorders (see **Table 1** for details).

**Table 1:** Characteristics of different types of prostatitis and healthy people.

Cohort characteristics	IIprostatitis (n=15)	IIIprostatitis (n=29)	Healthy people (n=13)
Age (yr)	33.0 (27.0-35.0)	31.0 (25.0-36.0)	29.0 (24.5-34.5)
Body mass index (kg/m^2^)	24.5 (20.5-27.1)	24.4 (21.0-26.5)	24.2 (21.5-26.0)
Smoking			
Yes	5/15 (33.3)	13/29 (44.8)	3/13 (23.1)
No	10/15 (66.7)	16/29 (55.2)	10/13 (76.9)
Drinking			
Yes	5/15 (33.3)	7/29 (24.1)	2/13 (15.4)
No	10/15 (66.7)	22/29 (75.9)	11/13 (84.6)
Chewing betel nut			
Yes	3/15 (20.0)	12/29 (41.4)	3/13 (23.1)
No	12/15 (80.0)	17/29 (58.6)	10/13 (76.9)
Hypertension			
Yes	2/15 (13.3)	2/29 (6.9)	0/13 (0.0)
No	13/15 (86.7)	27/29 (93.1)	13/13 (100.0)
NIH-CPSI score			
Pain symptom score	5.0 (1.0-7.0)	7.0 (4.0-10.0)	0.0 (0.0-4.5)
Urination symptom score	4.0 (3.0-8.0)	6.0 (4.5-8.0)	2.0 (1.0-5.0)
Quality of life score	8.0 (4.0-10.0)	9.0 (7.0-10.0)	5.0 (2.0-8.0)
Total symptom score	11.0 (6.0-13.0)	13.0 (10.0-16.0)	3.0 (1.5-7.0)
Total score	18.0 (12.0-22.0)	22.0 (17.0-26.0)	8.0 (5.0-14.5)
GAD-7 score	6.0 (4.0-10.0)	6.0 (4.0-13.0)	3.0 (0.0-6.0)
PHQ-9 score	4.0 (0.0-8.0)	9.0 (5.0-13.0)	5.0 (2.0-6.0)

IQR, interquartile range; Data are presented as n (%) or median (IQR); NIH-CPSI, The National Institutes of Health chronic prostatitis symptom index; GAD-7, Generalized Anxiexy Disorde-7; PHQ-9, Patient Health Questionnaire-9.

### 3.2 16S rRNA gene sequencing analysis of the microbial composition

#### 3.2.1 OTU abundance analysis

**Figure 1:**
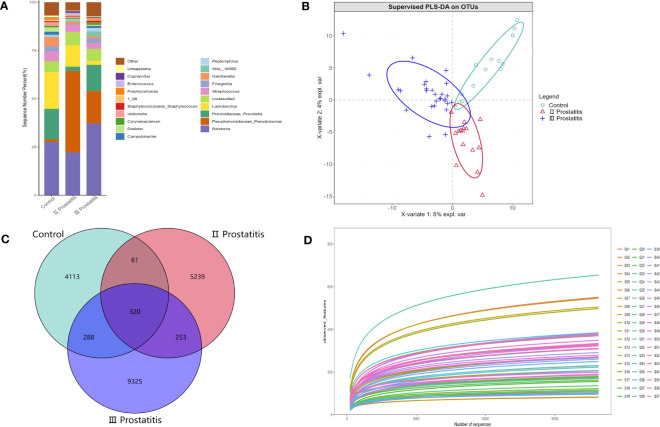
**(A)** The relative distribution of each group at the genus level (the species in the top 20 of relative abundance). The illustration shows the 20 most dominant species at the genus level, and the remaining species with relatively low abundance are classified as Other shown in the figure; **(B)** PLS-DA coordinate map. Each point represents a sample, the points of the same color belong to the same group, and the points of the same group are marked with an ellipse; **(C)** Wayne diagram based on OUT. Showing the common or unique number of OTU between different groups. Each ellipse represents a group; **(D)** Dilution curve of Alpha diversity index. The X axis extracts the sequence sequencing quantity in the graph, and the Y axis represents the corresponding Alpha diversity index. Each color represents a sample.

A total of 4,408,023 readable fragments were obtained from the data of 57 samples. After clustering/denoising all the effective sequences, 19,599 Operational Taxonomic Units (OTUs) were obtained. According to the unique or common OTUs among the samples, we draw a Venn diagram to visually show the composition similarity and overlap of the samples in each group at the OTU level (**Figure 1C**). Simultaneously, we used the R language mixOmics package to analyze all the OTUs with an abundance greater than 10 (**Figure 1B**). From the Venn diagram and the PLS-DA analysis coordinate diagram, it is easy to see many microorganisms in the EPS of different types of CP patients and healthy people. Still, they have their characteristics in abundance and structure, with some differences between groups.

#### 3.2.2 Analysis of taxonomic annotations

The species annotation information was obtained by comparing the representative OTU sequences with the Greengene database (Greengenes Database version 13_8). Our study annotated 36 phyla, 100 classes, 165 orders, 237 families, 432 genera, and 274 species. **Figure 1A** shows the relative distribution of species in the top 20 of relative abundance in different groups at the genus level. Additionally, **Figure S1** further details the relative distribution of species in the top 20 of relative abundance in each sample at the genus level.

#### 3.2.3 Heatmap analysis

**Figure 2:**
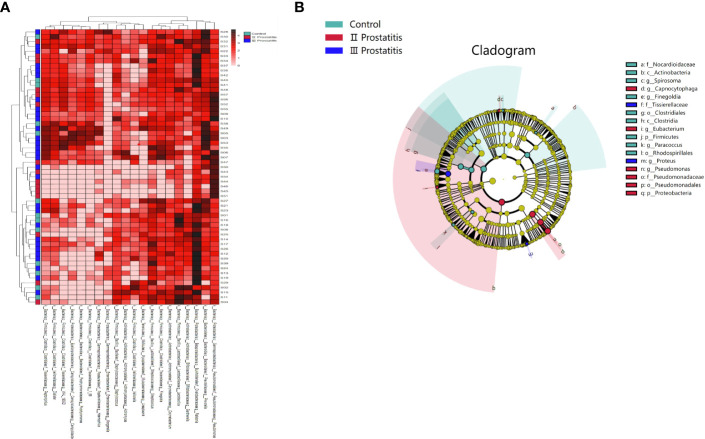
**(A)** A heat map of the genus level. Longitudinally is the sample name information. Horizontal is the annotation name of the genus horizontal classification. The clustering tree at the top of the figure is the similarity clustering of species abundance distribution in all samples. The clustering tree on the left is the similarity clustering of species abundance distribution of samples. The middle heat map is log10 (absolute abundance) heat map; **(B)** LEfSe analysis cladogram diagram (LDA>2). From inside to outside, cladogram diagrams correspond to different classification levels of families and genera, and the connections between levels represent the relationship of belonging. Each circle node represents a species. The yellow node signifies no significant difference between groups, and not yellow means that the species is the characteristic microorganism of the corresponding color group (the abundance is significantly higher in this group). The colored fan-shaped areas are marked with the sub-classification range of characteristic microorganisms.

To investigate the similarities or differences between samples and groups, horizontal clustering was carried out from two aspects of classification information and differences between samples to find the aggregation law of species or samples. The heatmap cluster analysis of all samples was carried out at the phylum, class, order, family, genus (**Figure 2A**), and species level. According to the result of the 16s rRNA sequence, the top five species of bacteria were *Streptococcus*, *Lactobacillus*, *Vibrio*, *Campylobacter*, and *Faecalibacterium* at the genus level.

#### 3.2.4 Sample-based rarefaction analysis

**Figure 3:**
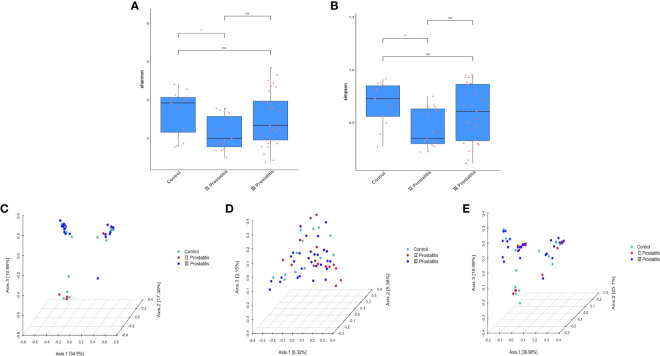
**(A)** Box diagram of Shannon index; **(B)** Box diagram of Simpson index; **(C)** 3D map of PCoA analysis based on Bray Curtis distance; **(D)** 3D map of PCoA analysis based on Unweighted Unifrac distance; **(E)** 3D map of PCoA analysis based on Weighted Unifrac distance. The asterisk (*) and double asterisks (**) represent p<0.05 and p<0.01, respectively.

The qiime2 diversity plug-in analyzed the Alpha diversity of the samples to evaluate the microbial diversity of the samples themselves. The index of the sparse curve of different Alpha diversity shows that the sequencing depth has covered all the species in the sample (**Figure 1D**). We describe microbial diversity by observed features, Chao, Faith’s Phylogenetic Diversity, Shannon, and the Simpson index. After obtaining the overall Alpha diversity index, combined with grouping information, the Wilcox Test was used to compare the significant differences between groups. The results showed that the Shannon (**Figure 3A**) and Simpson index (**Figure 3B**) had significant differences in flora diversity between type II prostatitis and healthy control groups. Beta diversity analysis was used to evaluate the differences in microbial community structure among samples. After Bray Curtis obtained the whole Beta diversity index, the microbial community structure between groups was compared by the PERMANOVA method, and the Beta diversity PCoA map (**Figures 3C–E**) and NMDC map (**Figures S2A–C**) were drawn. The results also showed significant differences between groups (**Table 2**).

**Table 2:** Results of Beta diversity analysis (pairwise permanova results).

Types	Group 1	Group 2	Sample size	Permutations	pseudo-F	p-value	q-value
Bray Curtis distance	Control	IIprostatitis	28	999	3.376	0.009	**0.017**
		IIIprostatitis	42	999	1.417	0.164	0.164
	IIprostatitis	IIIprostatitis	44	999	3.502	0.011	**0.017**
Unweighted Unifrac distance	Control	IIprostatitis	28	999	1.251	0.017	0.051
		IIIprostatitis	42	999	0.968	0.577	0.577
	IIprostatitis	IIIprostatitis	44	999	1.172	0.061	0.092
Weighted Unifrac distance	Control	IIprostatitis	28	999	3.107	0.016	**0.042**
		IIIprostatitis	42	999	2.038	0.076	0.076
	IIprostatitis	IIIprostatitis	44	999	2.957	0.028	**0.042**

q value is the corrected p value, which is of more reference value. Bold values indicate the statistically significant q values (q ≤ 0.05).

#### 3.2.5 Significance analysis of intergroup differences

**Figure 4:**
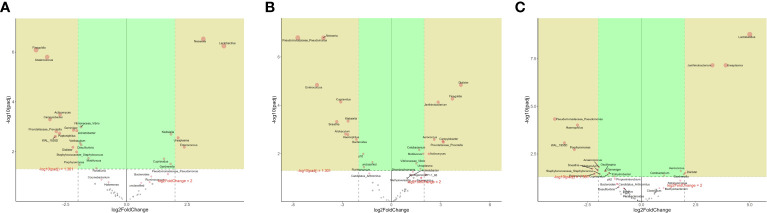
**(A)** DESeq2 analysis of volcano map in the genus level (numerator II prostatitis denominator III prostatitis); **(B)** DESeq2 analysis of volcano map in the genus level (numerator control denominator II prostatitis); **(C)** DESeq2 analysis of volcano map in the genus level (numerator control denominator III prostatitis).

The microbes with significant differences between groups were found using the R language DESeq2 package. Multiple comparisons between groups were carried out by the DESeq2 method (**Figure 2B**). The results showed that at the genus level, 15 kinds of characteristic microorganisms were found when the type II prostatitis group was compared with the type III prostatitis group, including 4 species in the type II prostatitis group (*Neisseria*, *Lactobacillus*, *Ureaplasma* and *Enterococcus*) and 11 species in the type III prostatitis group (*Finegoldia*, *Anaerococcus*, *Actinomyces*, *Campylobacter*, *Prevotellaceae Prevotella*, *Gemmiger*, *Acinetobacter*, *Peptoniphilus*, *WAL_1855D*, *Dialister* and *Staphylococcaceae Staphylococcus*). For details, see **Figure 4A** (log2FC > 2 and P < 0. 05). After comparing the type II prostatitis group with the healthy control group, 16 kinds of characteristic microorganisms were found, including 9 species in the type II prostatitis group (*Pseudomonadaceae Pseudomonas*, *Neisseria*, *Enterococcus*, *Cupriavidus*, *Klebsiella*, *Sneathia*, *Allobaculum*, *Haemophilus* and *Bacteroides*) and 7 species in the healthy control group (*Dialister*, *Finegoldia*, *Janthinobacterium*, *Aerococcus*, *Campylobacter*, *Prevotellaceae Prevotella* and *Actinomyces*). For details, see **Figure 4B** (log2FC > 2 and P < 0. 05). When comparing the type III prostatitis group with the healthy control group, 11 kinds of characteristic microorganisms were found, including 7 species in the type III prostatitis group (*Pseudomonadaceae Pseudomonas*, *Haemophilus*, *WAL_1855D*, *Porphyromonas*, *Sneathia*, *Allobaculum* and *Enterocecus*) and 4 species in the healthy control group (*Lactobacillus*, *Janthinobacterium*, *Ureaplasma* and *Dialister*). For details, see **Figure 4C** (log2FC > 2 and P < 0. 05). In our study, the LEfSe method was also used to detect the characteristic microorganisms in each group based on the relative abundance table (LDA > 4). The results showed that at the genus level, when LDA Score was equal to 4, 17 species of characteristic microorganisms were found, including 6 species in the type II prostatitis group, 2 species in the type III prostatitis group, and 9 species in the healthy control group.

### 3.3 Metagenomic analysis revealed different functional profiles

#### 3.3.1 Heatmap analysis

**Figure 5:**
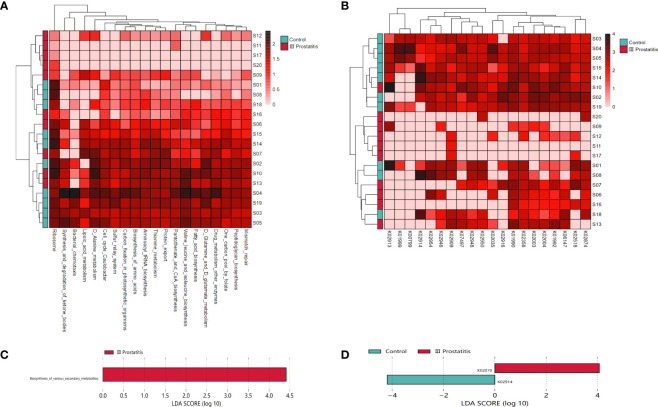
**(A)** Clustering heat map of KEGG metabolic pathway in each sample (the pathway in the top 20 of relative abundance); **(B)** Clustering heat map of KEGG ORTHOLOGY(KO) in each sample (relative abundance of the top 20 genes); **(C)** LDA histogram for LEfSe analysis of KEGG metabolic pathway (LDA>4); **(D)** LDA histogram for LEfSe analysis of KO genes (LDA>4).

To further study the function of microorganisms in EPS of CP patients, according to the results of 16s rRNA gene sequencing analysis, we selected 10 representative samples of microbial abundance in type III prostatitis group and healthy control group, carried out metagenomics sequencing, and annotated the metagenomics sequencing data in KEGG database (Videcnik et al., 2015; Peng et al., 2020). The heatmap cluster analysis was performed for relational results (**Figures 5A, B**). According to the results of metagenomics sequencing, the first five KEGG pathways involved were Ribosome (map03010), D Alanine metabolism (map00473), Cell cycle Caulobacter (map04112), Aminoacyl tRNA biosynthesis (map00970), and Biosynthesis of amino acids (map01230). The first five kinds of KOs involved were K01990, K02358, K02874, K01992, and K02909.

#### 3.3.2 Analysis of the difference in the relative abundance of function

**Figure 6:**
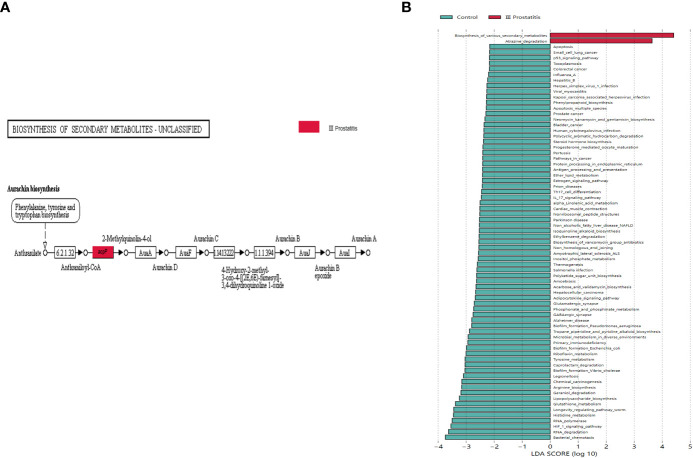
**(A)** Biomarkers genes in different groups of differential pathways. The gray rectangle indicates that the gene was detected in the sample, and the colored rectangle is the characteristic gene of the color corresponding grouping. **(B)** LDA histogram for LEfSe analysis of KEGG metabolic pathway (LDA>2).

The biomarkers of each group were found by the LEfSe analysis method combined non-parametric test and linear discriminant analysis. In terms of pathway level, two KEGG pathways were identified, which were significantly different in the type III prostatitis group (LDA > 2 and p < 0.05) (**Figure 6B**). Among them, the difference in the Aurachin biosynthesis (map00999) pathway under the biosynthesis of various secondary metabolites was the most significant, and this pathway also gathered the important KO: K02078 (LDA > 4, p < 0.05) (**Figures 5C, D**). According to the LEfSe differences between KO groups, we further colored the pathway map, marked the detected genes and the gene biomarkers of each group, and found that the acpP gene was significantly expressed in the samples of type III prostatitis (**Figure 6A**).

## 4 Discussion

16s rRNA and metagenomics sequencing techniques were used to detect and analyze the composition of microorganisms in EPS of patients with type II, type III prostatitis, and healthy people, and to explore the function of different bacteria in the grouping of type III prostatitis. The results showed that microorganisms were commonly found in the EPS of healthy people and patients with type II and III prostatitis. Compared with healthy people, there were the same types of different bacteria in EPS of patients with type II and type III prostatitis. Still, there were significant differences in Alpha and Beta diversity in patients with type II prostatitis. At the same time, there was no significant difference in Alpha and Beta diversity in patients with type III prostatitis. Most different bacteria in patients with type II and type III prostatitis overlap, but the relative abundance of type II is much higher than that of type III. At the same time, the results of metagenomics sequencing in patients with type III prostatitis showed that the different bacteria had certain functions. Therefore, we speculate that type III prostatitis may also be caused by infection with pathogenic bacteria and that patients with type III prostatitis can’t detect pathogenic bacteria from their EPS by bacterial culture *in vitro*, probably because the abundance of pathogenic bacteria is too low to reach the detection threshold.

Microbes are commonly detected in EPS of patients with type II, type III prostatitis, and healthy people (see **Figures 1A–C**), consistent with previous researchers’ perception of the ubiquity of microbes in human organs. The normal organs of the human body are not entirely sterile but host many microorganisms, which have unique flora structures and local microecology that may lead to some diseases when the flora is maladjusted (Fang, 2014; Cavarretta et al., 2017; Alfano et al., 2018). There were research reports that bacteria were isolated from normal prostate tissue, prostate cancer tissue, and EPS samples from patients with CP (Lee, 2015; Davidsson et al., 2016; Feng et al., 2019).

Compared with healthy people, the diversity of Alpha and Beta in patients with type II prostatitis showed significant differences (see **Figures 3A–E**; **Table 2**), consistent with the clinical characteristics of pathogenic bacteria in EPS of type II prostatitis by bacterial culture *in vitro*. As well as type II prostatitis, the relative abundance of *Pseudomonas*, *Haemophilus*, *Sneathia*, *Allobaculum*, and *Enterococcus* in EPS of patients with type III prostatitis was also significantly higher than that of healthy subjects (see **Figure 4C**). However, the relative abundance of *Pseudomonas* was much lower than that of patients with type II prostatitis (see **Figure 4A**). We speculate that pathogenic bacteria can’t be detected in patients with type III prostatitis during *in vitro* bacterial culture, probably because the absolute abundance of different bacteria in their EPS has not reached the lowest threshold of *in vitro* culture.

In addition, metagenomic analysis of typical type III prostatitis samples was conducted to explore the function of different bacteria (Raes et al., 2007). The KEGG function analysis showed that there were significant differences in two metabolic pathways between Atrazine degradation (map00791) and Aurachin biosynthesis (map00999) (LDA > 2) (**Figure 6B**). At the same time, the acpP (acyl carrier protein P) gene in Aurachin biosynthesis (map00999) metabolic pathway was significantly expressed in the type III prostatitis group (see **Figure 6A**). It has been confirmed that the acpP gene is the essential gene synthesized by the cell wall of opportunistic pathogens such as *Pseudomonas aeruginosa* and *Baumannii* (Nejad et al., 2021; Tekintas et al., 2021). Notably, many bioengineering studies focus on reducing the level of the acpP gene. From the 16s rRNA sequencing results of our research, it can be seen that *Pseudomonas* is the most significant difference between type II and type III prostatitis compared with healthy people (see **Figures 4B, C**). Therefore, we think the acpP gene’s high expression in type III prostatitis may be caused by the conditional pathogen *Pseudomonas*. As a common clinical conditional pathogen, *Pseudomonas* has the characteristics of low pathogenicity, but strong drug resistance and is widely distributed in normal skin, intestinal tract, and genitourinary tract (Vo et al., 2022). As one of the key regulatory genes in the Aurachin biosynthesis pathway, the acpP gene can participate in producing secondary metabolites of microorganisms. In the Aurachin biosynthesis pathway, the product aurachin D and related biosynthesis aurachin RE and aurachin SS have optimal antibacterial effects (Kitagawa et al., 2013; Zhang et al., 2017; Kruth et al., 2022).

When the body is fragile, conditional pathogenic bacteria produce secondary metabolite antibiotics to compete with other normal or beneficial bacteria for living space and resources and finally develop into the dominant bacteria of local microecology, resulting in clinical manifestations of the disease. For CP patients, when conditional pathogens develop into dominant strains and their absolute abundance is higher than the threshold needed for general bacterial culture *in vitro*, patients are diagnosed as type II prostatitis and type III when below the threshold (recessive infection). Simultaneously, because the local microecology of patients with CP has been stimulated by low concentrations of antibiotics (different bacterial metabolites) for a long time, producing a certain degree of resistance, the clinical efficacy of current drug treatments is not good.

This study has some noted limitations. First, due to the limitation of testing costs and other factors, the clinical sample size is small, and a larger cohort needs to be evaluated in future studies. Secondly, our conclusions are only based on 16s rRNA, metagenomics sequencing, and related research results. Thus, these findings need to be verified by further basic experiments. Of course, the above deficiencies are also the contents of our follow-up research.

## 5 Conclusions

Many pathogenic microorganisms exist in the EPS of CP patients and healthy people. *Pseudomonas*, *Haemophilus*, *Sneathia*, *Allobaculum*, and *Enterococcus* are different bacteria in EPS patients with type II and type III prostatitis. Functional analysis shows they may be the main culprits for prostatitis in CP patients. In traditional CP typing, both type II and type III should be related to bacterial infection, but the abundance of pathogenic bacteria in EPS differs. Notably, pathogenic microorganisms’ secondary metabolites may be one reason for the poor clinical drug treatment effect and prolonged course of disease in patients with CP, especially in patients with type III prostatitis.

## Data availability statement

The data presented in the study are deposited in the NCBI repository, accession number PRJNA982408.

## Ethics statement

The studies involving human participants were reviewed and approved by Institutional Review Board (IRB) of The Third Xiangya Hospital of Central South University (Grant No. 22069). The patients/participants provided their written informed consent to participate in this study.

## Author contributions

W-JS initiated the research question and supervised all aspects of the study; JG and J-WH contributed to data acquisition; YL supervised the statistical procedures of the manuscript; W-JS wrote the first version of the manuscript; ZL and L-YH reviewed this article. All authors contributed to the article and approved the submitted version.

## Supplementary material

Supplementary Figure 1 The relative distribution of each group at the genus level (the species in the top 20 of relative abundance). The illustration shows the 20 most dominant species at the genus level, and the remaining species with relatively low abundance are classified as Other shown in the figure.

Supplementary Figure 2
**(A)** NMDS Analysis based on Bray Curtis distance; **(B)** NMDS Analysis based on Unweighted Unifrac distance; **(C)** NMDS Analysis based on Weighted Unifrac distance; NMDS: Non-metric multidimensional scaling.
