# Supplementary material for: Is type III prostatitis also associated with bacterial infection?
Source: Front Cell Infect Microbiol. 2023 Jul 3;13:1189081. doi: 10.3389/fcimb.2023.1189081 (PMC10351278; doi:10.3389/fcimb.2023.1189081)
Supplement: Supplementary Figure 1 — The relative distribution of each group at the genus level (the species in the top 20 of relative abundance). The illustration shows the 20 most dominant species at the genus level, and the remaining species with relatively low abundance are classified as Other shown in the figure. [file DataSheet_1.doc]

**
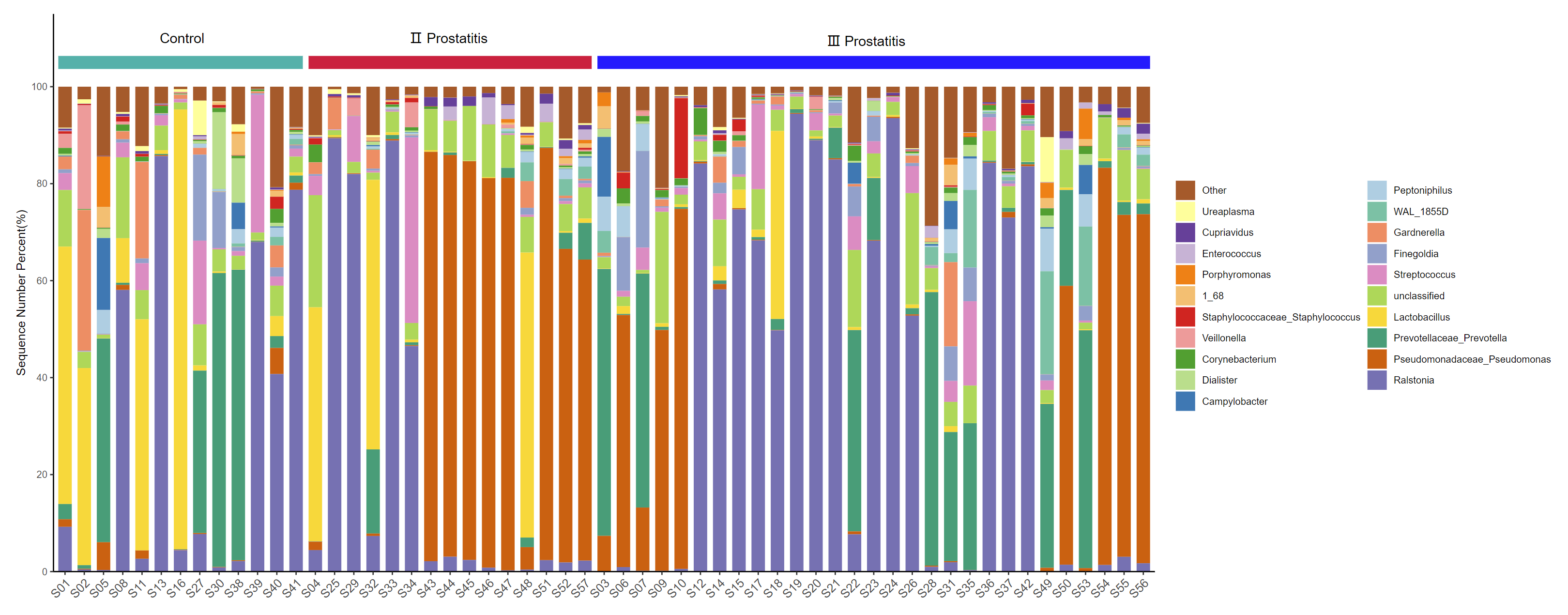
**


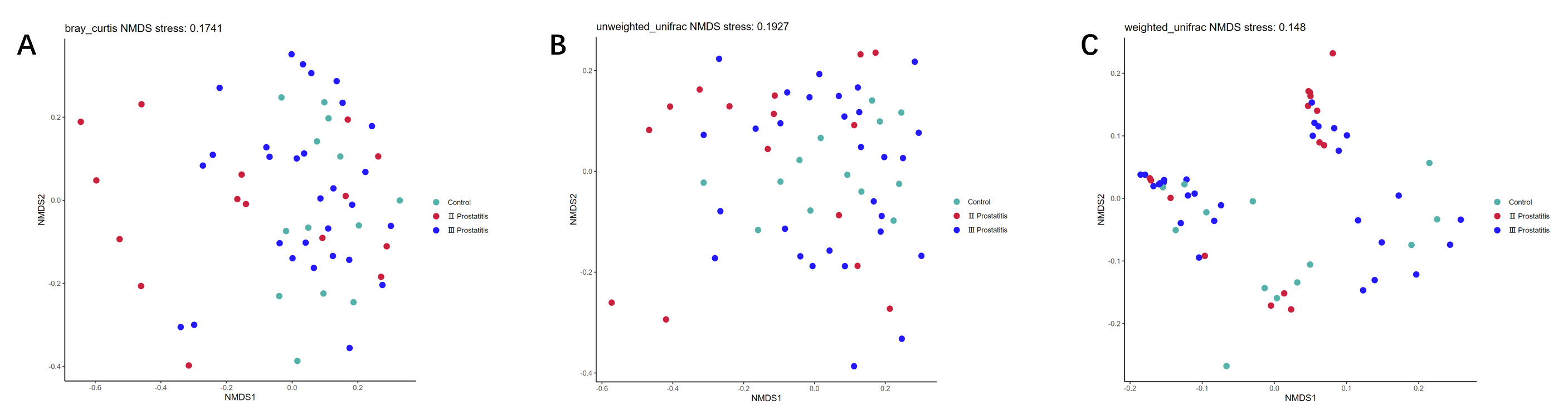
**FIGURE S1** The relative distribution of each group at the genus level (the species in the top 20 of relative abundance). The illustration shows the 20 most dominant species at the genus level, and the remaining species with relatively low abundance are classified as Other shown in the figure.

**FIGURE S2.** (A) NMDS Analysis based on Bray Curtis distance; (B) NMDS Analysis based on Unweighted Unifrac distance; (C) NMDS Analysis based on Weighted Unifrac distance; NMDS: Non-metric multidimensional scaling.

**Table S1** The National Institutes of Health chronic prostatitis symptom index (NIH-CPSI)

1. Which of the following areas have you experienced pain or discomfort in the past week?

a. Between the rectum (anus) and testis (scrotum) and perineum Yes (1 score) No (0 score)

b. Testis Yes (1 score) No (0 score)

c. The head of the penis (not related to urination) Yes (1 score) No (0 score)

d. Below the waist, bladder or pubic region Yes (1 score) No (0 score)

2. What have you been through in the last week?

a. Pain or discomfort when urinating? Yes (1 score) No (0 score)

b. Ejaculation hurts when or after the best part of sex. Yes (1 score) No (0 score)

3. How much time do you have any pain or discomfort?

Never (0 score) Very few (1 score) Sometimes (2 score)

Often (3 score) Usually (4 score) Always (5 score)

4. Which of the following numbers best describes your average pain or discomfort these days in the past week?

0 1 2 3 4 5 6 7 8 9 10

5. In the past week, how many times did you urinate after you finished urinating?

No (0 score) Less than 1/5 (1 score) Less than 1/2 (2 score)

Greater than 1/2 (3 score) More than 1/2 (4 score) Always (5 score)

6. In the past week, how many times did you urinate within 2 hours after you finished urinating?

No (0 score) Less than 1/5 (1 score) Less than 1/2 (2 score)

Greater than 1/2 (3 score) More than 1/2 (4 score) Always (5 score)

7. Have the above symptoms affected your daily life in the past week?

No (0 score) Just a little (1 score) Some (2 score) A lot of the time (3 score)

8. Have you always thought about your symptoms in the past week?

No (0 score) Just a little (1 score) Some (2 score) A lot of the time (3 score)

9. How do you feel if the symptoms of the past week are always with you for the rest of your daily life?

Very pleased (0 score) Happy (1 score) Most of the time satisfied (2 score)

Half satisfied and half dissatisfied (3 score) Dissatisfied most of the time (4 score) Unhappy (5 score) Terrible (6 score)

Total score:

**Table S2** Generalized Anxiexy Disorde-7 (GAD-7)

1. Feel uneasy, worried and irritable

No (0 score) For a few days (1 score) More than half the time (2 score) Almost every day (3 score)

2. Can't stop or can't control worry

No (0 score) For a few days (1 score) More than half the time (2 score) Almost every day (3 score)

3. Worry too much about all kinds of things

No (0 score) For a few days (1 score) More than half the time (2 score) Almost every day (3 score)

4. Very nervous, it's hard to relax

No (0 score) For a few days (1 score) More than half the time (2 score) Almost every day (3 score)

5. So anxious that can't sit still

No (0 score) For a few days (1 score) More than half the time (2 score) Almost every day (3 score)

6. To become easily annoyed or irritated

No (0 score) For a few days (1 score) More than half the time (2 score) Almost every day (3 score)

7. Feeling as if something terrible is going to happen.

No (0 score) For a few days (1 score) More than half the time (2 score) Almost every day (3 score)

Total score:

**Table S3** Patient Health Questionnaire-9 (PHQ-9)

1. Lack of motivation or interest in doing things

No (0 score) For a few days (1 score) More than half the time (2 score) Almost every day (3 score)

2. Feeling depressed, depressed, or desperate

No (0 score) For a few days (1 score) More than half the time (2 score) Almost every day (3 score)

3. Difficulty in falling asleep, restless or sleeping too much

No (0 score) For a few days (1 score) More than half the time (2 score) Almost every day (3 score)

4. Feeling tired or lack of energy

No (0 score) For a few days (1 score) More than half the time (2 score) Almost every day (3 score)

5. Lose appetite or eat too much

No (0 score) For a few days (1 score) More than half the time (2 score) Almost every day (3 score)

6. Feeling bad or feel like a failure, or let yourself or your family down

No (0 score) For a few days (1 score) More than half the time (2 score) Almost every day (3 score)

7. It is difficult to focus on things, such as reading newspapers or watching TV

No (0 score) For a few days (1 score) More than half the time (2 score) Almost every day (3 score)

8. Acting or speak so slowly that others are aware of it? Or just the opposite-become more irritable or restless than usual, moving around

No (0 score) For a few days (1 score) More than half the time (2 score) Almost every day (3 score)

9. It's better to die or hurt yourself in some way

No (0 score) For a few days (1 score) More than half the time (2 score) Almost every day (3 score)

Total score:
